# Multifaceted Natural Language Processing Task–Based Evaluation of Bidirectional Encoder Representations From Transformers Models for Bilingual (Korean and English) Clinical Notes: Algorithm Development and Validation

**DOI:** 10.2196/52897

**Published:** 2024-10-30

**Authors:** Kyungmo Kim, Seongkeun Park, Jeongwon Min, Sumin Park, Ju Yeon Kim, Jinsu Eun, Kyuha Jung, Yoobin Elyson Park, Esther Kim, Eun Young Lee, Joonhwan Lee, Jinwook Choi

**Affiliations:** 1 Interdisciplinary Program for Bioengineering, Seoul National University, Seoul, Republic of Korea; 2 Seoul National University Medical Research Center, Seoul, Republic of Korea; 3 Institute of Medical and Biological Engineering, Medical Research Center, Seoul National University, Seoul, Republic of Korea; 4 Division of Rheumatology, Department of Internal Medicine, Seoul National University Hospital, Seoul, Republic of Korea; 5 Human Computer Interaction and Design Lab, Seoul National University, Seoul, Republic of Korea; 6 Seoul National University College of Medicine, 103 Daehak-ro, Jongno-gu, Seoul, 03080, Republic of Korea, 82 2-766-3421

**Keywords:** natural language processing, NLP, natural language inference, reading comprehension, large language models, transformer

## Abstract

**Background:**

The bidirectional encoder representations from transformers (BERT) model has attracted considerable attention in clinical applications, such as patient classification and disease prediction. However, current studies have typically progressed to application development without a thorough assessment of the model’s comprehension of clinical context. Furthermore, limited comparative studies have been conducted on BERT models using medical documents from non–English-speaking countries. Therefore, the applicability of BERT models trained on English clinical notes to non-English contexts is yet to be confirmed. To address these gaps in literature, this study focused on identifying the most effective BERT model for non-English clinical notes.

**Objective:**

In this study, we evaluated the contextual understanding abilities of various BERT models applied to mixed Korean and English clinical notes. The objective of this study was to identify the BERT model that excels in understanding the context of such documents.

**Methods:**

Using data from 164,460 patients in a South Korean tertiary hospital, we pretrained BERT-base, BERT for Biomedical Text Mining (BioBERT), Korean BERT (KoBERT), and Multilingual BERT (M-BERT) to improve their contextual comprehension capabilities and subsequently compared their performances in 7 fine-tuning tasks.

**Results:**

The model performance varied based on the task and token usage. First, BERT-base and BioBERT excelled in tasks using classification ([CLS]) token embeddings, such as document classification. BioBERT achieved the highest *F*_1_-score of 89.32. Both BERT-base and BioBERT demonstrated their effectiveness in document pattern recognition, even with limited Korean tokens in the dictionary. Second, M-BERT exhibited a superior performance in reading comprehension tasks, achieving an *F*_1_-score of 93.77. Better results were obtained when fewer words were replaced with unknown ([UNK]) tokens. Third, M-BERT excelled in the knowledge inference task in which correct disease names were inferred from 63 candidate disease names in a document with disease names replaced with [MASK] tokens. M-BERT achieved the highest hit@10 score of 95.41.

**Conclusions:**

This study highlighted the effectiveness of various BERT models in a multilingual clinical domain. The findings can be used as a reference in clinical and language-based applications.

## Introduction

Since 2015, deep learning is increasingly being used in clinical natural language processing (NLP) [1]. Large language models (LLMs) based on deep learning technology are widely used in numerous clinical NLP domains [2]. Because contextual comprehension is critical for the overall performances of NLP models, studies have focused on the development of models that excel in conveying contextual information. Conventional approaches of NLP involve crafting word-to-word sequence models such as the hidden Markov model and using limited datasets annotated with labels such as disease and medication names [3-5]. However, studies are increasingly focusing on fine-tuning LLMs that have been pretrained on massive unlabeled biomedical literature sources, such as Medical Information Mart for Intensive Care (MIMIC-III) [6] and PubMed [7,8]. This shift in the NLP research direction has substantially elevated the contextual understanding capabilities of models and inspired studies on clinical NLP that focus on LLM utilization. For example, studies on automated summarization [9-11] have effectively extracted critical phrases from diverse sources, including biomedical papers and patient records. In addition, studies on entity extraction [12-15] have identified major entities such as disease names and drug names. However, these studies have focused exclusively on English-language corpora.

In the multilingual clinical domain, we proposed a set of contextual understanding conditions, with a comprehensive suite of clinical NLP evaluations specifically for these conditions. The proposed approach involves comparatively assessing bidirectional encoder representations from transformers (BERT) models [16] to provide guidelines for selecting the most suitable BERT model for a particular condition.

We proposed 2 hypotheses to examine 4 BERT models. First, we assumed that within the multilingual clinical domain, a language model capable of comprehending multiple languages would achieve superior performance. Second, models with the capacity to comprehend medical contexts would demonstrate superior efficacies. We selected BERT-base [16], Korean BERT (KoBERT) [17], Multilingual BERT (M-BERT) [18], and BERT for Biomedical Text Mining (BioBERT) [7] for the study. We pretrained these models on visit records on 160,000 patients. Subsequently, we introduced a series of comprehensive downstream tasks to learn the conditions required for these models to achieve effective contextual understanding. We assumed that an effective language model thrives in contextual comprehension under the following conditions:

- The model can determine whether the provided documents pertain to the same patient (tasks 1 and 2).
- The model is proficient in identifying the department associated with a given document (task 3).
- The model can discern the descriptions within medical records for the conditions of different patients (tasks 4 and 5).
- The model can ascertain the connection among sentences (task 6).
- The model can competently deduce disease names based on existing knowledge (task 7).

The rationale of the proposed conditions is the widespread adoption of BERT models in the medical domain for various applications.

BERT has been applied in medical natural language inference research to assess the relationship between 2 sequences (premise and hypothesis) with entailment, contradiction, or neutrality labels. Percha et al [19] used a fine-tuned BERT to locate clinical notes relevant to query sentences. Romanov and Shivade [20] created the MedNLI clinical dataset for natural language inference. They used several models and methodologies, such as bag-of-words, InferSent, and enhanced sequential inference models, to confirm the efficacy and validity of their datasets. Boukkour et al [21] introduced an alternative approach to BERT tokenization, proposing a convolutional neural network–based character-based tokenizer as a replacement for WordPiece Tokenizer, which is used to pretrain BERT, to improve BERT performance on the MedNLI dataset. Kanakarajan et al [22] pretrained the ELECTRA model, which is named “efficiently learning an encoder that classifies token replacements accurately,” [23] using abstracts from PubMed, and evaluated its performance on the MedNLI dataset.

BERT was applied to categorize clinical notes. Rasmy et al [24] introduced Med-BERT, which pretrained BERT using electronic health record data to classify diabetes and pancreatic cancer datasets. This model exceeded gated recurrent units by 2‐4 in terms of area under the receiver operating characteristic score. Zhang and Jankowski [25] proposed average pooling transformer layers handling token-, sentence-, and document-level embeddings for classifying *International Classification of Diseases* codes. Their model outperformed the BERT-base model by 11 points.

For the reading comprehension task, BERT can be used to determine the answer span within a given text. Pampari et al [26] proposed the electronic medical record question answering (emrQA) dataset to determine the answer span to a question in a clinical context. Yue et al [27] compared the performances of BERT-base, BioBERT, and ClinicalBERT [8] on the emrQA dataset and additional test datasets to address the problems of the emrQA dataset. Rawat et al [28] used 30 logical forms to express questions in semistructured texts and identified the correct responses in the emrQA dataset. They entered clinical notes and questions and used multitask training to simultaneously predict the logical structure of the question and the text span of the answer in a clinical note. Savery et al [29] introduced the MEDIQA-AnS dataset, which contains questions and corresponding answers regarding the health care concerns of patients. The correct answers to these questions, which contain valuable information about the patients, are used as summaries.

BERT can be used to extract information from clinical notes. Yang et al [15] used the 2010 i2b2 [30], 2012 i2b2 [31], and 2018 national NLP clinical challenges (n2c2) [32] datasets to compare the information extraction performances of BERT models, namely, BERT-base, ELECTRA, A Lite BERT (ALBERT) [33], and Robustly Optimized BERT Pretraining Approach (RoBERTa) [34]. The test results revealed that RoBERTa outperformed the other models. Richie et al [35] used Clinical BERT [8] to extract the social determinants of patient health, namely, employment, living tobacco, alcohol, drug use, and their attributes, from the n2c2 2022 Track 2 dataset [36]; for instance, texts such as “works” and “unemployed” were extracted for detailing employment information.

Although studies have extensively examined BERT versatility, they have focused only on English corpora. To address this limitation, we comprehensively analyzed the efficacies of BERT models in various tasks involving medical documents in both Korean and English.

The rest of the manuscript is organized as follows. The *Methods* section outlines the diverse tests used for BERT analysis and their application procedures. The *Results* section presents a summary of the outcomes of each test. The *Discussion* section outlines the distinctive characteristics of each BERT model and presents a thorough analysis for understanding the reasons for these characteristics. Finally, the *Conclusion* section summarizes the study and emphasizes its significance.

The aim of this study was to identify the BERT models that perform optimally in the bilingual (Korean and English) clinical domain. To achieve this objective, we designed 7 tasks, evaluated the performance of 4 BERT variants (BERT-base, BioBERT, KoBERT, and M-BERT) across these tasks, and assessed their relative significance.

## Methods

### Dataset

We obtained outpatient records from 8 departments, namely, endocrinology, respiratory, cardiovascular, gastroenterology, rheumatology, nephrology, allergy medicine, and infectious medicine departments, at Seoul National University Hospital in South Korea. We collected the records of 164,460 outpatients between 2010 and 2019. The dataset comprised 2,453,934 documents, with 412,499,140 tokens generated after tokenization using white space. The distribution of tokens and documents for various departments was as follows: endocrinology (tokens: 91,352,271; docs: 496,938), respiratory (tokens: 31,556,578; docs: 195,048), cardiovascular (tokens: 114,978,554; docs: 696,061), gastroenterology (tokens: 57,755,571; docs: 416,062), rheumatology (tokens: 24,857,675; docs: 204,600), nephrology (tokens: 70,865,514; docs: 322,629), allergy medicine (tokens: 17,024,481; docs: 92,041), and infectious medicine departments (tokens: 4,108,496; docs: 30,555). Table 1 provides statistical data for the corpus. Table 2 presents the clinical note of a patient experiencing rheumatoid arthritis.

**Table 1.** Statistical data of clinical notes in Seoul National University Hospital between 2010 and 2019.

Department	Tokens, n	Documents, n
Endocrinology	91,352,271	496,938
Respiratory	31,556,578	195,048
Cardiovascular	114,978,554	696,061
Gastroenterology	57,755,571	416,062
Rheumatology	24,857,675	204,600
Nephrology	70,865,514	322,629
Allergy medicine	17,024,481	92,041
Infectious medicine	4,108,496	30,555
Sum	412,499,140	2,453,934

**Table 2.** The example of a clinical note that was used for training bidirectional encoder representations from transformers models (for better understanding, an English translation has been added).

Section	Contents
History	Korean: *3117.2.1 arthralgia r/o d/t letrozole 로 병원 방문*; English (translated): *3117.2.1 arthralgia, rule out (r/o) due to letrozole. Visited hospital* Korean: *meloxicam 7.5 mg bid 복용한 hx 있다.*; English (translated): *Has a history of taking meloxicam 7.5 mg twice daily.* Korean: *f/u loss 마지막 방문 때 RF*^a^*, ACCP*^b^*, ANA*^c^ *등 처방했다*.; English (translated): *Prescribed RF, ACCP, ANA, etc, during the last visit.* Korean: *Arthralgia , neutropenia 가 있다. 손이 붓고 마디가 아프다. 약먹지만 붓기가 빠지지 않는 것 같다.*; English (translated): *Experiencing arthralgia and neutropenia. Hands are swollen and joints are painful. Although taking medication, the swelling does not seem to be going down.*
P/E & Lab^d^	Korean: *PIP S −/+ T −/− wrist S −/+ , T −/+ Toe s −/− T −/− 2120 . 3 lab bone scan: normal CBC*^e^*, WNL*^f^*, and CRP*^g^ *0.10*; English (translated): *PIP S −/+ T −/− wrist S −/+ , T −/+ Toe s −/− T −/− 2120 . 3 lab bone scan: normal CBC, WNL, and CRP 0.10*
Assessment	Korean: *Arthralgia r/o d/t letrozole 장상피화생*; English (translated): *Arthralgia r/o d/t letrozole. Intestinal metaplasia*
Plan	Korean: *RF, ACCP, ANA , x-ray Celebrex 50 mg tid -->Celebrex 100 mg tid*; English (translated): *RF, ACCP, ANA, x-ray Celebrex 50 mg tid -->Celebrex 100 mg tid*

a RF: rheumatoid factor.

b ACCP: anticitrullinated protein antibody.

c ANA: antinuclear antibody.

d P/E & Lab: physical examination and laboratory.

e CBC: complete blood count.

f WNL: within normal limits.

g CRP: C-reactive protein.

### Ethical Considerations

We obtained approval to use the original data collection for research purposes from the institutional review board (IRB) at Seoul National University Hospital (IRB no. C-2108-008-1242). According to the institution’s IRB policy, the data cannot be publicly disclosed due to patient privacy concerns. Instead, we provide an overview of the data in Table 2.

### BERT Models

The BERT-base model is a precursor in pretrained transformer encoders [37]. Vast open-domain data sources, including Wikipedia and BooksCorpus, are used to train the model [38]. The model is primarily focused on English text. The configuration of this dataset facilitates the expression of contextual representations of English sequences.

The BioBERT model is an evolution of BERT and is pretrained on PubMed data and enriched with biomedical entities, rendering BioBERT proficient in comprehending terminologies such as disease and drug names. In this study, we used the latest iteration of BioBERT, that is, BioBERT version 1.1.

The SKT Corporation in South Korea devised the KoBERT model to enhance the comprehension and processing of the Korean language. Data from Korean Wikipedia and news articles were used to pretrain the model.

The M-BERT model was obtained from a richly varied corpus of 104 languages, enabling a contextual representation that spans both English and Korean sequences.

### Pretraining

To enhance the bilingual clinical contextual understanding capabilities of BERT models, we conducted additional pretraining using an extensive dataset comprising 159,460 out of 164,460 outpatient records from Seoul National University Hospital, employing masked language modeling. The data were preprocessed meticulously using this strategy. WordPiece Tokenizer was used by BERT-base, BioBERT, and M-BERT; SentencePiece Tokenizer [39] was used by KoBERT. All tokenizers were case-sensitive. Subsequently, random tokens within the input sequence were replaced with [MASK] tokens. This process was reiterated 10 times to yield the data required for pretraining. The pretraining task of the model involved reinstating the [MASK] token to its original token, drawing on the data crafted through this preprocessing procedure.

### Multifaceted Clinical NLP Tasks

The evaluation framework encompassed 5 characteristics. Each characteristic was examined through 7 distinct downstream tasks that were designed to assess the clinical contextual comprehension capabilities of various BERT models.

#### Homogeneity Determination

**Figure 1.**
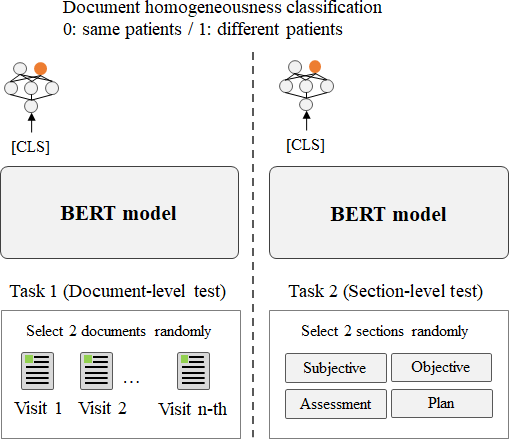
Document homogeneousness test (tasks 1 and 2). BERT: bidirectional encoder representations from transformers; CLS: classification.

As seen in Figure 1, we used 2 single outpatient records per input sequence to determine document homogeneity. Each model performed binary classification, discerning whether the records corresponded to those of the same patient (task 1). We extended this examination to the section level, tasking each model with predicting homogeneity based on a smaller segment of a page (task 2). In task 2, the objective was to determine whether 2 sequences originated from the same patient record, with 1 sequence containing an assessment section and the other section containing a randomized section.

#### Document Representativeness

**Figure 2.**
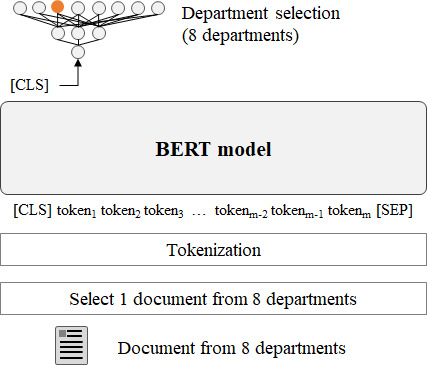
Document representativeness test: classifying documents (task 3). BERT: bidirectional encoder representations from transformers; CLS: classification; SEP: separator.

As seen in Figure 2, to assess document representativeness, we devised a task that focused on department identification by using individual visit records (task 3).

#### Reading Comprehension Test

**Figure 3.**
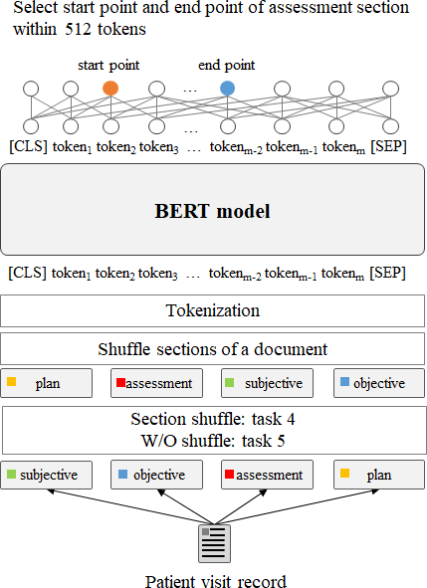
Reading comprehension test: identifying the department associated with a given document (with section shuffling: task 4; w/o section shuffling: task 5). BERT: bidirectional encoder representations from transformers; CLS: classification; SEP: separator; w/o: without.

The reading comprehension test (Figure 3) test extracted summarized content from a visit record. We focused on extracting the assessment section from the Subjective, Objective, Assessment, Plan (SOAP) or the history, physical examination, laboratory, assessment, and plan sections. The experiments encompassed 2 setups, namely, 1 setup with section-shuffled documents (task 4) and 1 setup with maintained section-order documents (task 5).

#### Contextual Connections

**Figure 4.**
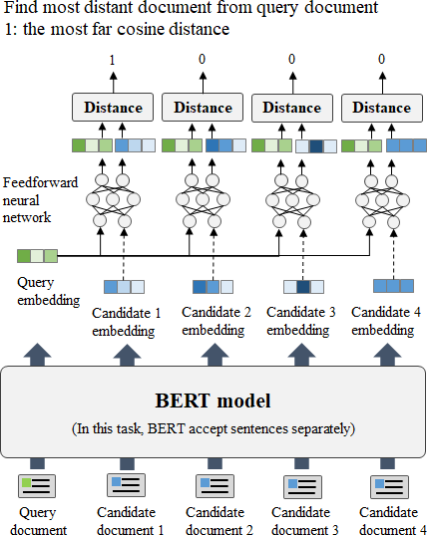
Document connectivity test: finding the last visited document (task 6). BERT: bidirectional encoder representations from transformers.

As seen in Figure 4, we introduced a task that required the model to differentiate the most recent visit record from a set of 4 candidate documents when given a query document representing the oldest visit record (task 6). The limitation of BERT models regarding the amount of the input length they can handle necessitates a workaround because simultaneously inputting both the query document and 4 candidate documents is not feasible. To address this problem, we adopted a 2-step approach. First, each individual document was independently inputted into BERT to acquire document embeddings. Subsequently, these document embeddings, forming a pair comprising the query document and the kth candidate document embeddings, were introduced into a feedforward neural network (FFNN) [40]. For example, if the query and document embedding pair for the most recent visit were inputted into the FFNN, the model was trained to output a prediction value of 1; this value was assigned based on our assumptions. We postulated that the query document, which corresponded to the earliest visit among the 5 documents, and the last document, which denoted the most recent visit, encompassed the most distinct narrative. Consequently, we measured the cosine distances between these 2 embeddings and directed the model to output a prediction value of 1, which indicated the greatest distance in terms of cosine similarity. By contrast, if the query and nonanswer document embedding pairs were presented to the FFNN, the model was trained to output a prediction value of zero.

#### Knowledge Reasoning

**Figure 5.**
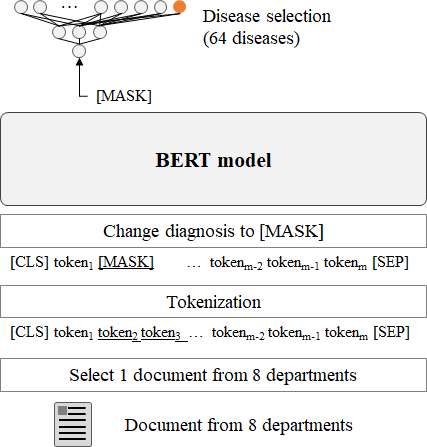
Knowledge reasoning test: finding the disease name (task 7). BERT: bidirectional encoder representations from transformers; CLS: classification; SEP: separator.

The knowledge reasoning characteristic (Figure 5) evaluated the capacity of a model to deduce entities from masked text (task 7). Each model was tasked with deducing disease names from masked visit records in which the disease names had been replaced with [MASK] tokens. We used MetaMap [41] to create a dataset by identifying diagnostic names. Each model, when presented with the [MASK] token and context, selected the correct disease name from 63 disease names. A comprehensive list of the entities is shown in Table S1 in Multimedia Appendix 1.

### Experimental Settings

We trained and evaluated 4 types of publicly available BERT models through the following process. We used records of 159,460 patients out of 164,460 patients for pretraining. In the pretraining procedure, 15% of random tokens from the 159,460 patient records were masked. Among them, 80% of the masked tokens were replaced with [MASK] tokens, 10% were replaced with random tokens, and the remaining 10% retained their original tokens. We trained the BERT models to restore [MASK] tokens to their original tokens.

After pretraining, the 4 BERT models were fine-tuned for tasks 1‐7. For fine-tuning, we used 5000 patient records that were not used in pretraining. We assigned 4000 patients to the training set and 1000 patients to the test set and then created training and evaluation data specific for each task. In each task, the 4 pretrained BERT models were trained using the training set and evaluated on the test set.

In the pretraining step, 4 NVIDIA 3090 graphics processing units (GPUs) were used in parallel for 3 epochs. After pretraining, all the models were fine-tuned using a 1080ti GPU except for task 6, in which 3090 GPU were used, because this task required more calculation procedures and memory. The detailed hyperparameter settings are described in Table S2 in Multimedia Appendix 1. The detailed experimental settings and analysis code used in this study are available on GitHub [42].

## Results

### Results of Tasks 1-3

In tasks 1‐3, BERT-base and BioBERT exhibited the best scores; Tables3  4 present the corresponding results.

**Table 3.** Results of various BERT^a^ models in tasks 1 and 2.

Model	Task 1: Determination of whether 2 documents are from the same patients			Task 2: Determination of whether 2 sections are from the same patients		
	Precision	Recall	*F*_1_-score	Precision	Recall	*F*_1_-score
BERT-base	84.44	94.19	89.05	89.28	87.60	88.43
BioBERT^b^	83.36	96.21	89.32	92.92	82.73	87.53
KoBERT^c^	83.95	74.05	78.69	90.68	75.78	82.56
M-BERT^d^	83.22	94.02	88.29	83.56	93.38	88.19

a BERT: bidirectional encoder representations from transformers.

b BioBERT: BERT for Biomedical Text Mining.

c KoBERT: Korean BERT.

d M-BERT: Multilingual BERT

**Table 4.** Results of various BERT^a^ models in task 3.

Model	Task 3: Identification of the department associated with a given document accuracy
BERT-base	96.75
BioBERT^b^	97.44
KoBERT^c^	95.38
M-BERT^d^	96.06

a BERT: bidirectional encoder representations from transformers.

b BioBERT: BERT for Biomedical Text Mining.

c KoBERT: Korean BERT.

d M-BERT: Multilingual BERT.

In the homogeneity test conducted on document-level inputs (task 1), BioBERT achieved the highest *F*_1_-score, whereas in the test conducted on the section-level inputs (task 2), BERT-base achieved the highest *F*_1_-score. Comparing the scores under tasks 1 and 2 revealed that BioBERT exhibited a more substantial drop in performance than those of other models. By contrast, KoBERT consistently demonstrated a diminished performance compared with that exhibited by other BERT models. In the document representativeness test, which entailed the selection of a single department from a set of 8 department candidates, BioBERT exhibited superior performance in terms of accuracy, which was the evaluation metric.

### Results of Tasks 4-7

In tasks 4‐7, M-BERT achieved the best scores (Tables5  6).

**Table 5.** Results of various BERT^a^ models in tasks 4 and 5.

Model	Task 4: Finding the assessment section with inputs that are section-shuffled			Task 5: Finding the assessment section with inputs that are not section-shuffled		
	Precision	Recall	*F*_1_-score	Precision	Recall	*F*_1_-score
BERT-base	71.03	61.74	60.83	74.59	59.14	56.69
BioBERT^b^	72.16	56.31	51.64	74.71	55.99	51.17
KoBERT^c^	76.57	77.41	76.88	92.61	93.88	93.15
M-BERT^d^	93.15	94.61	93.77	96.52	96.37	96.44

a BERT: bidirectional encoder representations from transformers.

b BioBERT: BERT for Biomedical Text Mining.

c KoBERT: Korean BERT.

d M-BERT: Multilingual BERT.

**Table 6.** Results of various BERT^a^ models in task 7.

Model	Task 7: Determination of disease names based on existing knowledge		
	hit@1	hit@3	hit@10
BERT-base^a^	60.26	77.47	93.40
BioBERT^b^	59.40	80.20	95.12
KoBERT^c^	46.20	72.02	91.54
M-BERT^d^	61.12	81.64	95.41

a BERT: bidirectional encoder representations from transformers.

b BioBERT: BERT for Biomedical Text Mining.

c KoBERT: Korean BERT.

d M-BERT: Multilingual BERT.

In the reading comprehension tests (tasks 4 and 5), the performances of the models were evaluated in terms of the *F*_1_-score, which was calculated by measuring the proportion of tokens within the predicted interval that correctly overlapped with the actual interval. M-BERT achieved the highest performance in reading comprehension tests. In addition, the models exhibited the largest performance differences in these tests. In the context connectivity test (task 6), M-BERT exhibited the highest performance with an *F*_1_-score of 64.75, whereas all the other models achieved a score lower than 60 (BERT-base: 59.78; BioBERT: 58.39; KoBERT: 25.62; and M-BERT: 64.75). In the knowledge-reasoning test (task 7), the M-BERT model exhibited the best performance. The primary objective of this test was to accurately prognosticate 63 potential candidate diagnoses, as extracted from clinical documents, in which the diagnosis name was substituted with [MASK]. In our assessment, we used hit@k (where *k*=1, 3, or 10). For instance, in task 7, BERT computes probabilities for 63 diseases based on a provided context. In this context, hit@k is a true positive if k diseases with the highest probability encompass the correct disease. The final evaluation score is then determined by dividing the number of true positives by the total number of sequences under assessment.

## Discussion

### Suitability of BERT-Base and BioBERT for English [CLS] Embedding (Tasks 1-3)

In tasks 1‐3, the BERT classification ([CLS]) embedding was the input for the FFNN. The [CLS] token, positioned at the far-left side of the input sequence, is a classification token. The embedding of this token is commonly used as a feature for classification tasks, indicating the model’s comprehension of segment-level or document-level context. In tasks 1 and 2, homogeneity was assessed at the document and section levels, respectively, and BioBERT and BERT-base demonstrated the highest performances, respectively. In task 3, BioBERT achieved the highest score. Based on these observations, we inferred that BERT-base and BioBERT would be suitable for tasks involving [CLS] embedding.

Generally, a model’s ability to understand context diminishes as the number of tokens absent from its dictionary increases. Unknown ([UNK]) tokens represent tokens absent from the model’s dictionary, and the presence of these tokens correlates with lower model performance. The higher the frequency of [UNK] tokens, the greater the challenge for the model to accurately comprehend the context. Notably, despite the limited inclusion of Korean tokens, these models excelled in tasks 1‐3 (Table S4 in Multimedia Appendix 1). BERT-base and BioBERT, which were pretrained on English sentence patterns, exhibited improved performances because of the prevalence of English sentences in outpatient visit records, which typically detailed their diseases.

### Influence of Multilingual Capabilities in Reading Comprehension Tasks on Outcomes (Tasks 4 and 5)

In tasks 4 and 5, the reading comprehension ability of the model was assessed by determining the scope of the assessment section. Among models, M-BERT demonstrated the highest performance, whereas BERT-base and BioBERT exhibited the lowest test scores. The presence of extensive multilingual capabilities in the reading comprehension tests was the predominant factor influencing these outcomes.

To comprehend why BERT-base and BioBERT exhibit markedly inferior performance compared with M-BERT in tasks 4 and 5, understanding the composition of the BERT model dictionaries and the function of the [UNK] token is crucial. In BERT models, a dedicated tokenizer is used to segment text into tokens. These tokens are retained if present in the model’s dictionary; otherwise, the tokens are substituted with [UNK] tokens, representing unknown entities. Consequently, a higher prevalence of [UNK] tokens indicates a diminished ability of the model to comprehend the semantic nuances of the sequence. In tasks 4 and 5, where each token’s semantic relevance determines its association with an assessment section, models with inadequate knowledge of individual tokens exhibit poor performance. The dictionaries of BERT-base and BioBERT contain minimal Korean characters, resulting in the majority of Korean tokens being replaced with [UNK] tokens. By contrast, M-BERT encompasses a comprehensive range of Korean characters in its dictionary. Therefore, BERT-base and BioBERT exhibit notably inferior performance in tasks 4 and 5 compared with M-BERT.

### Relationship Between Multilingual Capability and Task Complexity (Task 7)

Task 7, which was focused at evaluating the aptitude of a model for knowledge inference, was more complex than other tasks. Notably, M-BERT outperformed the other models in task 7, securing hit@1, hit@3, and hit@10 scores of 61.12, 81.64, and 95.41, respectively. These results highlighted the pivotal role of the dictionary in knowledge inference. Furthermore, when processing documents in multiple languages, M-BERT outperformed BERT-base, which had been exclusively trained in a single language.

For task 6, the test results were poor. An analysis indicated that BERT models did not excel in this task because of the prevalence of outpatient medical records in the copy-and-paste format (Table S6 in Multimedia Appendix 1). Consequently, the significance of task 6 in this study was low.

### Contributions to the Clinical Text Processing and Medical Fields

#### Importance of Multilingual Models

The experiment highlights the significance of using multilingual language models in processing bilingual clinical notes. The findings demonstrated that using a model capable of handling 2 languages yields superior performance compared with relying solely on a single language model. This insight is particularly relevant for countries such as Korea and Japan, where clinical documentation typically involves a mixture of languages.

#### Base for Model Selection

Furthermore, this study provides empirical evidence for choosing a proper BERT model, a factor not substantiated in existing NLP research. For instance, in previous studies, such as that conducted by Kim and Lee [43], M-BERT was used for tasks such as extracting disease names, symptoms, and body parts from Korean text without providing explicit justification. The experimental results satisfied this gap by showcasing the superiority of M-BERT in understanding bilingual clinical text and supporting appropriate BERT selection in future studies.

### Limitations and Future Works

#### Limited Scope of Clinical Notes

This analysis primarily focused on outpatient visit records. Future studies should encompass a broad range of clinical notes, including surgical notes, hospitalization records, and discharge summaries. Comparing and validating the performance of BERT models across various types of clinical documentation provides a comprehensive understanding of their effectiveness.

#### Single-Institution Data

This study exclusively used data from Seoul National University Hospital, which can limit the generalizability of the findings. Clinical notes can vary considerably in style and content across various health care institutions. Therefore, future studies should involve data from multiple hospitals to validate BERT model performance in various clinical settings.

#### More Tasks Should Be Verified

The BERT model requires further validation in bilingual clinical text. Oh et al [44] conducted a study to recognize protected health information in the publicly available i2b2 2014 dataset. However, we could not perform this task because manual labeled annotations are required to extract non-English entities in bilingual clinical notes. In future studies, various tasks using bilingual clinical notes should be proposed.

### Conclusions

In this study, we comprehensively compared 4 BERT models, encompassing text in both English and Korean, within the multilingual clinical domain. We pretrained these models with approximately 160,000 patient records and evaluated their performances for 7 diverse downstream tasks. The experimental findings are summarized as follows.

First, the BERT-base and BioBERT models excelled in document classification tasks using [CLS] tokens. These results highlighted their superiority over M-BERT in tasks involving simple pattern recognition in word sequences. Second, the significance of having a comprehensive dictionary was evident in the reading comprehension task in which comprehensive token usage was required. The exceptional performance of M-BERT, which encompassed a broad range of Korean and English tokens, clearly confirmed the importance of the dictionary. Third, multilingual proficiency was pivotal for tasks that demanded complex reasoning. Both M-BERT and BioBERT excelled in task 7, which focused on diagnosing a multitude of candidates, and notably, M-BERT consistently outperformed BioBERT.

Our findings highlighted the suitability of BioBERT and BERT-base for tasks that relied on sequence patterns in multilingual clinical domains. In addition, M-BERT, which had an expansive dictionary and aptitude for leveraging Korean and English clinical contexts, was highly suitable for tasks involving textual content comprehension. The experimental results of the BERT models in mixed-language clinical documents provide valuable insights for future medical NLP research and appropriate BERT model selection for different types of tasks.

## Supplementary material

10.2196/52897 Multimedia Appendix 1 Supplementary materials on disease entities, hyperparameter settings, number of documents in the pretraining dataset, tokens, tokenization, and masked language modeling loss.

## Abbreviations

[CLS]: classification
[UNK]: unknown
ALBERT: A Lite BERT
BERT: bidirectional encoder representations from transformers
BioBERT: BERT for Biomedical Text Mining
ELECTRA: efficiently learning an encoder that classifies token replacements accurately
emrQA: electronic medical record question answering
FFNN: feedforward neural network
GPU: graphics processing unit
HMM: hidden Markov model
IRB: institutional review board
KoBERT: Korean BERT
LLM: large language model
M-BERT: Multilingual BERT
MIMIC-III: Medical Information Mart for Intensive Care
NLP: natural language processing
RoBERTa: Robustly Optimized BERT Pretraining Approach
SOAP: Subjective, Objective, Assessment, Plan
