# Supplementary material for: Multifaceted Natural Language Processing Task–Based Evaluation of Bidirectional Encoder Representations From Transformers Models for Bilingual (Korean and English) Clinical Notes: Algorithm Development and Validation
Source: JMIR Med Inform. 2024 Oct 30;12:e52897. doi: 10.2196/52897 (PMC11539635; doi:10.2196/52897)
Supplement: Multimedia Appendix 1 [file medinform-v12-e52897-s001.docx]

**Supplementary Material Table S1**

**Supplementary Material Table S1.** Disease entities used in Knowledge Inference test (task 7).

| **UMLS  Concept id** | **disease name** | **UMLS  Concept id** | **disease name** |
| --- | --- | --- | --- |
| C0020538 | Hypertensive disease | C0263428 | Burnett Schwartz Berberian syndrome |
| C0029456 | Osteoporosis | C0017661 | IGA Glomerulonephritis |
| C0011389 | Dental Plaque | C0242528 | Azotemia |
| C0242339 | Dyslipidemias | C3887499 | Renal cyst |
| C1561643 | Chronic Kidney Diseases | C0085413 | Polycystic Kidney, Autosomal Dominant |
| C0015695 | Fatty Liver | C2316810 | Chronic kidney disease stage 5 |
| C0032027 | Pityriasis Rubra Pilaris | C1956415 | Paroxysmal nocturnal dyspnea |
| C0677607 | Hashimoto Disease | C0024117 | Chronic Obstructive Airway Disease |
| C0020676 | Hypothyroidism | C0521530 | Lung consolidation |
| C0029453 | Osteopenia | C0024115 | Lung diseases |
| C0002170 | Alopecia | C0034067 | Pulmonary Emphysema |
| C0270724 | Infantile Neuroaxonal Dystrophy | C0006267 | Bronchiectasis |
| C0149745 | Oral Ulcer | C0032285 | Pneumonia |
| C0029408 | Degenerative polyarthritis | C0004096 | Asthma |
| C0018099 | Gout | C1832594 | Verloes Bourguignon syndrome |
| C0024141 | Lupus Erythematosus, Systemic | C0009450 | Communicable Diseases |
| C0042164 | Uveitis | C0020473 | Hyperlipidemia |
| C0151636 | Premature ventricular contractions | C1535939 | Pneumocystis jiroveci pneumonia |
| C0242231 | Coronary Stenosis | C0020557 | Hypertriglyceridemia |
| C0020443 | Hypercholesterolemia | C0152244 | Bone Cysts, Aneurysmal |
| C0393706 | Early infantile epileptic encephalopathy  with suppression bursts | C0004135 | Ataxia Telangiectasia |
| C0010054 | Coronary Arteriosclerosis | C0041296 | Tuberculosis |
| C0036916 | Sexually Transmitted Diseases | C0019360 | Herpes zoster disease |
| C0031039 | Pericardial effusion | C0019693 | HIV Infections |
| C0019196 | Hepatitis C | C0039128 | Syphilis |
| C1112705 | Nuclear non-senile cataract | C2607914 | Allergic rhinitis (disorder) |
| C0010709 | Cyst | C0008711 | Chronic rhinitis |
| C0003962 | Ascites | C1861559 | CHROMATE RESISTANCE (disorder) |
| C3887938 | Deuteranomaly | C0037199 | Sinusitis |
| C0267834 | Liver cyst | C0263338 | Chronic Urticaria |
| C0015674 | Chronic Fatigue Syndrome | C0035455 | Rhinitis |
|  |  | C4551720 | Primary Ciliary Dyskinesia |

**Supplementary Material Table S2**

**Supplementary Material Table S2.** Hyperparameter settings in tasks.

| **Task** | **batch  size** | **learning rate** | **warmup  proportion** | **Epoch** | **Machine** | **# train** | **# test** |
| --- | --- | --- | --- | --- | --- | --- | --- |
| Pretrain^1)^ | 16 | 0.00002 | 0.85 | 10^2)^ | Nvidia RTX 3090 × 4 | 60,940,880 | - |
| Task 1 | 8 | 0.00003 | 1.00 | 2 | Nvidia RTX 1080ti × 1 | 7,990 | 2,349 |
| Task 2 | 8 | 0.00003 | 1.00 | 2 | Nvidia RTX 1080ti × 1 | 12,340 | 3,084 |
| Task 3 | 8 | 0.00003 | 1.00 | 2 | Nvidia RTX 1080ti × 1 | 8,000 | 1,600 |
| Task 4 | 8 | 0.00003 | 1.00 | 2 | Nvidia RTX 1080ti × 1 | 11,492 | 150 |
| Task 5 | 8 | 0.00003 | 1.00 | 2 | Nvidia RTX 1080ti × 1 | 11,492 | 150 |
| Task 6 | 40 | 0.00003 | 1.00 | 2 | Nvidia RTX 3090 × 4^3)^ | 100,615 | 3,220 |
| Task 7 | 8 | 0.00003 | 1.00 | 2 | Nvidia RTX 1080ti × 1 | 2,316 | 697 |

^1)^ Pretraining Objective function was Masked Language Modeling.
^2)^ We set 10 epochs, however, halted pretraining when epoch 3, because it took about six days for three epochs.
We pretrained four BERT models, therefore total pretraining time took around 20 days.  ^3)^ We finetuned models on four 3090 GPU cards in task 6 because the task required the models to calculate more than 2 times the procedures than other tasks. For instance, in task 1, the model accepted concatenated two truncated documents within 512 lengths. Unlike task 1, in task 6, the model accepted two documents separately. Then, the model calculated cosine distance using 2 document embeddings.

**Supplementary Material Table S3**

**Supplementary Material Table S3.** Average number of documents per patents in pretraining dataset.

| **Department** | **average number of docs. per patient** |
| --- | --- |
| infectious medicine | 6.73 |
| endocrinology | 10.60 |
| rheumatology | 14.50 |
| gastroenterology | 6.18 |
| cardiovascular | 12.14 |
| nephrology | 13.48 |
| allergy medicine | 9.44 |
| respiratory | 6.21 |
| macro average | 9.91 |

**Supplementary Material Table S4**

**Supplementary Material Table S4.** Occupation ratio of [UNK] tokens within test sets.
BERT-base, BioBERT used the same dictionary because BioBERT was further pretrained from BERT-base parameters. BERT-base, BioBERT showed the highest [UNK] token occupation in all dataset, because those models didn't handle Korean in the vocabulary well. Nonetheless, BERT-base, BioBERT exhibited the highest score in tasks 1-3. However, this lack of word coverage range caused relatively low scores than M-BERT in tasks 4-7.

| Models | Ratio | PT* | Task 1 | Task 2 | Task 3 | Tasks 4  and 5 | Task 6 | Task 7 |
| --- | --- | --- | --- | --- | --- | --- | --- | --- |
| BERT-base | %[UNK] total | **10.66** | **9.88** | **11.30** | **10.36** | **7.57** | **8.65** | **9.36** |
|  | %[UNK] in en | **0.17** | **0.13** | **0.12** | **0.16** | **0.08** | **0.16** | **0.15** |
|  | %[UNK] in ko | **98.79** | **99.29** | **99.60** | **99.53** | **99.31** | **99.46** | **99.46** |
| BioBERT | %[UNK] total | **10.66** | **9.88** | **11.30** | **10.35** | **7.57** | **8.65** | **9.36** |
|  | %[UNK] in en | **0.17** | **0.13** | **0.12** | **0.16** | **0.08** | **0.16** | **0.15** |
|  | %[UNK] in ko | **98.79** | **99.29** | **99.60** | **99.53** | **99.31** | **99.46** | **99.46** |
| KoBERT | %[UNK] total | 0.14 | 0.10 | 0.12 | 0.13 | 0.10 | 0.15 | 0.13 |
|  | %[UNK] in en | 0.15 | 0.11 | 0.14 | 0.15 | 0.11 | 0.16 | 0.14 |
|  | %[UNK] in ko | 0.06 | 0.00 | 0.00 | 0.00 | 0.00 | 0.00 | 0.00 |
| M-BERT** | %[UNK] total | 0.07 | 0.05 | 0.06 | 0.07 | 0.06 | 0.06 | 0.06 |
|  | %[UNK] in en | 0.06 | 0.03 | 0.03 | 0.03 | 0.01 | 0.05 | 0.05 |
|  | %[UNK] in ko | 0.10 | 0.13 | 0.15 | 0.22 | 0.30 | 0.13 | 0.08 |

*PT: Pretraining dataset, **M-BERT: Multilingual BERT

**Supplementary Material Table S5**

**Supplementary Material Table S5.** Examples of Tokenization.
BERT-base, BioBERT, and M-BERT used WordPiece Tokenizer. KoBERT used SentencePiece tokenizer. Since BioBERT and KoBERT only provide case-sensitive versions, we used case-sensitive versions of BERT-base and M-BERT for fair comparison.

| **Type** | **Expression** |
| --- | --- |
| Source | 백내장 수술을 하였음 |
| Translation | Had cataract surgery |
| BERT_base (Cased) | [UNK] [UNK] [UNK] |
| M-BERT (Cased) | 백 ##내 ##장 수 ##술 ##을 하 ##였 ##음 |
| BioBERT (Cased) | [UNK] [UNK] [UNK] |
| KoBERT (Cased) | 백 내 장 수술 을 하 였 음 |

**Supplementary Material Table S6**

**Supplementary Material Table S6.** Disease entities used in Knowledge Inference test (task 7).
Since we couldn’t disclose the patient's original medical records, we have described fictitious documents rewritten based on real documents. We used data that had not been translated into English in our experiments.

| **Input type** | **document** |
| --- | --- |
| Query (3115-04-20) | **[History]** He is doing regular exercise. **[P / E & Lab .]** AST ( GOT ) 15, ALT ( GPT ) 20, IDMS MDRD GFR 108, Creatinine 0.66, hs - CRP 1.11, ESR 37 **[Assessment]** ]He suffered side effects from the etanercept medication, so he stopped taking it. He is suffering from high blood pressure. A mild abnormality was observed in his liver, but it has now improved. He had tuberculosis five years ago, but was cured. Lately he has been suffering from osteoporosis and his thigh muscles have shrunk. **[Plan]** I will prescribe airtal medication. |
| Candidate 1 (3115-07-20) | **[History]** He had pain in his all feets at night, and he couldn't straighten his feets. **[P / E & Lab .]** AST ( GOT ) 28, ALT ( GPT ) 22, Creatinine 0.55, IDMS MDRD GFR 81, hs - CRP 0.33, ESR 0.33 **[Assessment]** He suffered side effects from the etanercept medication, so he stopped taking it. He is suffering from high blood pressure. A mild abnormality was observed in his liver, but it has now improved. He had tuberculosis five years ago, but was cured. Lately he has been suffering from osteoporosis and his thigh muscles have shrunk. |
| Candidate 2 (3116-03-20) | **[History]** He continues to suffer from the symptoms of shriveled feet. **[P / E & Lab .]** ESR 33, AST ( GOT ) 28, ALT ( GPT ) 30, Creatinine 0.84, IDMS MDRD GFR 88, hs - CRP 0.22 **[Assessment]** He suffered side effects from the etanercept medication, so he stopped taking it. He is suffering from high blood pressure. A mild abnormality was observed in his liver, but it has now improved. He had tuberculosis five years ago, but was cured. Lately he has been suffering from osteoporosis and his thigh muscles have shrunk. He is suspected of having nephropathy. He has a bladder prolapse and is going to have surgery. |
| Candidate 3 (3121-11-22) | **[History]** There is a persistent symptom of shriveled feet. There is no pain. Recently, He started exercising for his legs. **[P / E & Lab .]** ESR 15, AST ( GOT ) 22, ALT ( GPT ) 22, Creatinine 0.60, hs - CRP 0.11 **[Assessment]** He suffered side effects from the etanercept medication, so he stopped taking it. He is suffering from high blood pressure. A mild abnormality was observed in his liver, but it has now improved. He had tuberculosis five years ago, but was cured. Lately he has been suffering from osteoporosis and his thigh muscles have shrunk. He is suspected of having nephropathy. He has a bladder prolapse and is going to have surgery. **[Plan]** I explained to the patient about pain control with celecoxib. |
| Candidate 4 (3123-05-15) | **[History]** He gets cramps when he sleeps at night. He is scheduled to undergo surgery for a cystocele. **[P / E & Lab .]** ESR 33, hs - CRP 0.05 **[Assessment]** He suffered side effects from the etanercept medication, so he stopped taking it. He is suffering from high blood pressure. A mild abnormality was observed in his liver, but it has now improved. He had tuberculosis five years ago, but was cured. Lately he has been suffering from osteoporosis and his thigh muscles have shrunk. He is suspected of having nephropathy. He has a bladder prolapse and is going to have surgery. **[Plan]** Decided to continue taking celecoxib |

**Supplementary Material Figure S1**

| **MLM loss of BERT-Base (Cased)** | **MLM loss of BioBERT** |
| --- | --- |
| **MLM loss of KoBERT** | **MLM loss of M-BERT (Cased)** |

Supplementary Material Figure S1. MLM Loss of BERTs.
Batchsize for pretraining was 16, and the accumulation step was set to one. When the batchsize was increased by increasing the accumulation step value by more than one, a gradient explosion occurred. Since it took about two days to implement pretraining one epoch per model, we trained each model three epochs out of 10 epochs. Therefore, the total pretraining time was about 20 days.
